# Pathways of Photosynthesis in Non-Leaf Tissues

**DOI:** 10.3390/biology9120438

**Published:** 2020-12-02

**Authors:** Robert J. Henry, Agnelo Furtado, Parimalan Rangan

**Affiliations:** 1 Queensland Alliance for Agriculture and Food Innovation, University of Queensland, Brisbane, QLD 4072, Australia; a.furtado@uq.edu.au (A.F.); R.Parimalan@icar.gov.in (P.R.); 2 Division of Genomic Resources, ICAR-National Bureau of Plant Genetic Resources, New Delhi 110012, India

**Keywords:** photosynthesis, stem, seed, petiole, flower, C_3_ photosynthesis, C_4_ photosynthesis, CAM, respiration

## Abstract

**Simple Summary:**

Plants have leaves that are specialized organs to capture light energy. This energy is used to support photosynthesis, a process in which carbon dioxide from the atmosphere is incorporated into organic compounds in the plant to allow the plant to grow. Other parts of the plant, such as the stem, flowers, or seeds are also able to conduct photosynthesis to contribute to growth in many plant species. The main contribution of photosynthesis in these parts of the plant may be to use carbon dioxide produced by the plant in respiration rather than from the surrounding atmosphere. The biochemical processes used by the plant in these organs may be different from those used in the leaves of the same plant. This process is enhanced in plants under stress and may be important for plant survival in some situations. Increased knowledge of these processes may be used to develop plant varieties that are more tolerant of environmental extremes and could help adapt agriculture to climate change.

**Abstract:**

Plants have leaves as specialised organs that capture light energy by photosynthesis. However, photosynthesis is also found in other plant organs. Photosynthesis may be found in the petiole, stems, flowers, fruits, and seeds. All photosynthesis can contribute to the capture of carbon and growth of the plant. The benefit to the plant of photosynthesis in these other tissues or organs may often be associated with the need to re-capture carbon especially in storage organs that have high respiration rates. Some plants that conduct C_3_ photosynthesis in the leaves have been reported to use C_4_ photosynthesis in petioles, stems, flowers, fruits, or seeds. These pathways of non-leaf photosynthesis may be especially important in supporting plant growth under stress and may be a key contributor to plant growth and survival. Pathways of photosynthesis have directionally evolved many times in different plant lineages in response to environmental selection and may also have differentiated in specific parts of the plant. This consideration may be useful in the breeding of crop plants with enhanced performance in response to climate change.

## 1. Introduction

Plant leaves display a great diversity of shapes and forms but all share a common purpose. Leaves are specialized plant organs designed to capture light energy by photosynthesis. This energy drives the fixation of carbon from the atmosphere in photosynthesis. However, not all photosynthesis in plants is restricted to leaves. Photosynthesis in non-leaf tissues is found in many plants but the role of this photosynthesis is not well studied or understood. Here we examine the likely functions and mechanisms of photosynthesis in plant tissues other than those in the specialist structure of the leaf. Photosynthesis in non-leaf tissues may be important for growth and crop yield [1] especially under stress [2]. While the importance of non-leaf photosynthesis has been recognized, the biochemical pathways of photosynthesis in the tissues have received relatively little attention compared to those in the leaf. Diverse evidence suggests the functioning of different pathways in non-leaf tissues. The pathways may have some similarity to those in leaves but may also differ in detail.

## 2. Sites of Non-Leaf Photosynthesis

Examples of photosynthesis can be found in most if not all parts of plants (Table 1). Photosynthesis in the leaf blade is expected as this is a plant organ in flowering plants (angiosperms) designed for this purpose. Photosynthesis, in many plants, extends to the petioles [3], or leaf stalks, stems [3] (including branches or trunks), floral structures [4], seeds [5,6], and fruits [7,8] (Table 1). Plant parts that are not normally exposed to light such as roots or tubers can also become photosynthetic when exposed to light [9]. Photosynthesis in these tissues or organs may be seasonal, expressed at specific developmental stages, or in response to environmental triggers. Scientific understanding of the molecular and genetic regulation of photosynthesis in these tissues might facilitate the modification of plants to have enhanced carbon-fixation processes. The breeding of plants with increased capacity to conduct effective photosynthesis in non-leaf tissues might provide significant improvements in yields especially in environments where abiotic or biotic stresses limit yield. Climate change is likely to increase the frequency of the impact of these limitations that pose a major threat to food security [10,11]. In some plants, photosynthesis may be found throughout almost the whole plant. For example, tomatoes may conduct photosynthesis in the leaves, petioles, stems, and fruits [11]. Little evidence is available on the morphological characteristics of the chloroplast in non-leaf tissues. However, in the wheat endosperm, the grana in the chloroplasts of the cross-cells are stacked and there is less stacking in the tube-cells suggesting a dimorphism like that found in classical C_4_ plants [12].

### 2.1. Stems

The stems of plants are photosynthetic in some species [3]. Even the trunks of some tree species are photosynthetic [13,14]. Stem photosynthesis (corticular or woody tissue photosynthesis) has been shown to contribute to bud growth on defoliation as reported in *Prunus, Umbellularia,* and *Arctostaphylos* species, and shading of the trunk and defoliation was reported to reduce growth in these species. Defoliation resulted in ^13^C enrichment in sugars in the trunk phloem. This enrichment is consistent with the carbon being sourced from respiration [13]. Respired CO_2_ is known to be depleted in ^13^C [15]. The analysis of ^13^C abundance has been used to determine the pathways of photosynthesis in many plants. However, the complex pathway of carbon assimilation in many non-leaf tissues makes this analysis difficult as many biochemical steps, with the potential for carbon isotope discrimination, are involved in these pathways [16,17,18]. Carbon fixation in leaves (C_3_) followed by translocation to the seed, release in respiration, recapture by PEP carboxylase in internal tissues decarboxylation in outer photosynthetic tissues, and re-capture by rubisco make this a long pathway for analysis of carbon isotope discrimination. 

Trees with photosynthetic stems have been shown to have high nighttime sap flow [19,20]. However, the physiological basis of this has not been defined. Stem photosynthesis may be important in plants with few leaves at some development stages or under specific seasonal or environmental (e.g., water stress) conditions.

The stem and leaf-sheath of wheat were reported to photosynthesize and contribute around 10% of the grain weight in wheat [21]. Combining it with spike photosynthesis, where the estimated contribution to grain weight may be 40% [22], this suggests that nearly 50% of the grain weight in wheat cultivars maybe due to contributions through photosynthesis from non-leaf tissues (stem, leaf-sheath, and spikes). This underscores the importance of the photosynthetic contribution from non-leaf tissues, as potential targets of research in improving yield and productivity, especially in cereal crops.

The trunks of some Eucalypts are often green. Cadaghi (*Corymbia torelliana*) [23], a uniquely rainforest species, from this large plant group has apparently photosynthetic stems. This may be an adaptation to the high competition for light in the rainforest. Species of *Brachychiton* (Malvaceae) [24] have very thick trunks that appear to be very photosynthetic. Some of these species defoliate in the dry part of the year (rather than in the cold) as an apparent adaption to water stress. Photosynthesis in the trunk and branches may support the carbon needs of the plant when without leaves. In sugarcane, photosynthesis has been widely studied in the leaves but many photosynthesis genes are also expressed in the culms [25]. More work is required to assess the significance of non-leaf photosynthesis and to determine the biochemical processes involved in stem photosynthesis.

### 2.2. Petioles

Petioles are often photosynthetic [3] in tissues that are continuous with the leaf and connect to the stem. However, photosynthesis in petioles may be very different [26]. The petiole may be a structure with a low surface area to volume ratio resulting in a high proportion of the cells being internal (not exposed to light and unable to conduct photosynthesis) and releasing carbon dioxide in respiration. This low surface area to volume ratio is also characteristic of photosynthetic, stems, seeds, and fruits. The photosynthetic cells in surface layers overlay a very large number of internal cells that will be producers of CO_2_ in respiration. The cells surrounding the xylem and phloem of the petioles receive sugars from the vascular system as a source of carbon for photosynthesis in these tissues [3]. Hibberd and Quick [3] reported C_4_ photosynthesis in the petioles and stems of tobacco and celery. This process allows the capture of respired carbon dioxide by PEP carboxylase activity in these tissues.

### 2.3. Flowers

Flowers may have parts that are photosynthetic [27], including the petals and more often the sepals. Photosynthesis in these flower organs in *Encelia* species (Asteraceae) was reported to be higher at the bud stage and to compensate for the loss of carbon in respiration associated with the production of the reproductive structures [4] Giant ragweed (*Ambrosia trifida*) has been shown to produce around half of the carbon for reproductive structures in the flower [28]. Photosynthesis in sepals or other parts of flower buds may contribute to the capture of carbon respired during production of the flower tissues within the closed bud. Reproduction in seed plants depends upon successful formation of seeds and will be under strong evolutionary selection pressure. Supply of carbon for seed development is critical to reproductive success.

### 2.4. Seeds

Leaves are generally source tissues and seeds sink tissues in carbon capture and storage. Photosynthesis in the leaf (a source tissue) captures carbon that is translocated to sink tissues such as seeds (a sink tissue). The carbon export involves carbohydrates passing through many cells by either apoplastic or symplastic loading processes [29,30]. Photosynthesis in seeds complicates this perspective when the seed becomes both the site of carbon fixation and the site of accumulation or storage. 

The anatomy of seeds differs between species, resulting in different tissues having variable photosynthetic potential. The cotyledons are often photosynthetic in seeds of plants such as legumes with enlarged cotyledons such as in pea [31] and chickpea [32]. In addition to the cotyledons being photosynthetic during seed development, they may also be photosynthetic during the germination process. In hypogeal species, the cotyledons remain below the surface but in epigeal species, the cotyledons emerge as the first photosynthetic leaves. In soybean cotyledons, photosynthesis has been shown to compensate for the respiratory losses during germination [33]. Members of angiosperm families [5] such as the Amaryllidaceae possess a chlorophyllous endosperm or embryo or integument [34]. 

The major part of seeds of grasses such as wheat is the endosperm. The endosperm (the major seed storage tissue of cereals) is surrounded by the pericarp which is in turn enclosed in the glumes. Both the glumes and the pericarp are green and photosynthetic in nature. Both the tube cell and cross cell layers in the outer seed are highly photosynthetic and have surrounding tissues that are transparent allowing high levels of light penetration. Studies of photosynthesis in the wheat seed have involved analysis of different components of this system possibly explaining the diversity of reports about “ear” photosynthesis [35]. The pericarp has very few stomata [36] suggesting that the photosynthesis in this tissue is more likely to be involved in the re-capture of respired carbon from the seed. The pericarp has two photosynthetic cell layers, the tube and cross cells and these have morphologically distinct chloroplasts that could support a C_4_ type photosynthesis [12]. The pericarp is a bright green color when observed by removing the glumes at mid seed development at the height of grain filling. The glumes surrounding the seed have many more stomata and have specialized Kranz cells around the vascular bundles suggestive of the potential for a type of C_4_ photosynthesis in these cells [36]. This role of seeds in photosynthesis may be important at specific points in time in the life cycle of the plant in seed development or germination [37].

### 2.5. Fruits 

Photosynthesis can also be found in fruits [38] usually before ripening but sometimes also in the mature fruit. Familiar examples would be green tomatoes or the outer parts of other unripe fruit [39]. In the cucumber fruit, re-fixation of respired carbon makes an important contribution to growth and this involves PEP carboxylase [7]. Fruit often remain photosynthetic (green) until late in development when all growth is completed and when a change in color may be associated with the attraction of vectors for seed dispersal.

### 2.6. Roots

Most roots are not photosynthetic but some groups of plants have roots that are exposed to light. Epiphytes including orchids are good examples. Photosynthesis in roots has characteristics typical of those of other non-leaf tissues. Photosynthesis in *Vanda* roots has been shown to be activated by wetting [40]. CAM photosynthesis is found in these plants as evidenced by diurnal cycling of acid levels [50]. The Photosynthetic pathways in roots may differ from those in leaves [51].

## 3. Source of Carbon for Non-Leaf Photosynthesis

The source of carbon for photosynthesis in leaves is usually considered to be atmospheric CO_2_ entering the leaf via the stomata. Respiration is a major source of CO_2_ especially in deep tissues to which atmospheric CO_2_ cannot easily penetrate. Other tissues may be provided with carbon via the vascular system of the plant. Respiration has been suggested as the major source of carbon for seed photosynthesis [52]. In the wheat ear, photosynthesis was found to contribute between 10% and 44% of the grain yield depending upon the environment with re-fixation of respired carbon being a significant contributor to this grain yield [52]. The CO_2_ produced in respiration will become bicarbonate in these tissues and a substrate for PEP carboxylase. The pH in the grain remains high supporting bicarbonate stability until germination when rapid acidification is associated with the germination process [53].

Respiration is likely to be the major source of carbon for photosynthesis originating in non-leaf tissues deep within the plant in stems, seeds, or fruit and being captured by light drive reactions on the surface of the plant organ.

## 4. Dark Reactions of Photosynthesis in Non-Leaf Organs and Tissues

### 4.1. C3

The Calvin cycle or C_3_ pathway is common to all plants. This process involves a reaction between CO_2_ (a single carbon atom) and ribulose-1,5 bi-phosphate (5 carbon atoms) to produce 3-phosphoglyceric acid (with 3 carbon atoms). This is called C_3_ photosynthesis because the first products in the capture of CO_2_ are 3-carbon compounds as opposed to 4-carbon compounds in C_4_ photosynthesis. The components of this pathway are found in all photosynthetic tissues including non-leaf tissues active in photosynthesis. This pathway has been widely studied and is relatively well understood.

Some plants adapt to the carbon capture limitations of their environment by adding further steps to concentrate the carbon at the point of fixation by the ribulose-1,5-biphosphate carboxylase oxygenase (rubisco) of the C_3_ pathway. These enhanced pathways include C_4_ and CAM photosynthesis. These pathways or versions of them may be found in non-leaf tissues.

### 4.2. C4

The C4 pathway of photosynthesis has been reported in non-leaf tissues in plants that employ C_3_ photosynthesis in the leaf (Table 2). The C_4_ pathway fixes carbon initially in a 4 carbon compound to provide a method of carbon concentration to supply the carbon required for the C_3_ pathway [54]. This pathway involves an initial capture of carbon by phosphoenolpyruvate (PEP) carboxylase to form a 4 carbon compound (oxaloacetic acid). Oxaloacetic acid is converted to malic acid by the action of malate dehydrogenase. This is subsequently de-carboxylated by a malic enzyme (expressed specifically in the light) resulting in CO_2_ enrichment surrounding rubisco.

A diversity of types of C_4_ photosynthesis pathways are recognized in plants [55]. Different types of C_4_ photosynthesis have been defined based upon the presence of different decarboxylases (NAD-malic enzyme; NADP-malic enzyme and PEP carboxykinase) that are involved in decarboxylation reaction in different species. This reflects the different evolutionary origins of C_4_ photosynthesis in plants [56]. There has been little analysis of these differences in non-leaf tissues. However, a NAD-malic enzyme has been reported in wheat pericarp [12]. Recently, a non-Kranz NAD-ME subtype C_4_ photosynthesis within epidermal and mesophyll cells of the leaves of *Ottelia* was reported [57].

Debate about the pathways of photosynthesis in wheat seeds was recently revived by the discovery [56] of a separate group of genes encoding C_4_ specific enzymes and expressed specifically in the pericarp of the grain. This has resulted in re-examination of earlier research in this area [26,56]. There is evidence for C4 pathways in developing wheat seeds coming from labelling studies, metabolite profiles, enzyme specificity analysis, patterns of gene expression, and genome sequence analysis.

Early pulse labelling studies had traced the path of the carbon through malate to sugars in the isolated barley pericarp [44]. Some labelling experiments that have fed carbon to the whole wheat ear are difficult to interpret [58]. Some of these experiments provided labelled CO_2_ to the intact plant testing photosynthesis in the glumes and awns that are, like wheat leaves, well-characterized as predominantly C_3._ The incorporation of exogenous CO_2_ in C_3_ photosynthesis in these experiments has led to some skepticism of existence of C_4_ pathways in the seed. Photosynthesis in the pericarp relies overwhelmingly on respired carbon and is not so easily tested in experiments using exogenous CO_2_ with intact plants.

Early studies of enzyme activity in wheat showed that the enzymes in the wheat ear showed C_4_ characteristics. Analysis of gene expression in the developing wheat grain showed expression of genes encoding C_4_ photosynthesis in mid seed development [12] corresponding to the period of peak grain filling and maximal respiration to support starch and storage protein accumulation in the endosperm [45]. One misconception is that all genes necessary for C_4_ photosynthesis are also present in C_3_ genomes. C_4_ reactions rely on the presence of specialized versions of these genes. In the evolutionary process, gene or genome duplication events followed by neo-functionalization events are key factors responsible for gene variants with distinct specificities in C_3_ and C_4_ plants, and the same gene copies are co-opted across species for the accomplishment of C_4_ photosynthesis. For example, it has long been known that wheat expresses PEP carboxylase but Rangan et al. [12] showed that the seed expresses a distinct C_4_ form of the gene while the leaves express a conventional C_3_ form. The wheat C_4_ PEP carboxylase has a similar sequence to the maize C_4_ PEP carboxylase and is distinct from C_3_ type PEP carboxylases. These different PEP carboxylases in wheat are encoded by genes located on different chromosomes. These studies suggest that this tribe, the Triticeae, within the grasses may have active C_4_ photosynthesis in the pericarp but not in other parts of the plant. Many other grasses (e.g., sorghum) have developed C_4_ photosynthesis in the leaves.

The patterns of expression of C_4_ enzymes in the developing wheat seed are consistent with the production of PEP for the capture of respiratory carbon in the endosperm thereby feeding further fixation process through the C_4_ pathway in the cross- and tube-cell layers of the pericarp. Both pyruvate orthophosphate dikinase (PPDK) and rubisco were expressed in the green pericarp [59]. The timing of expression corresponds with the peak of grain filling. Malate has been reported to accumulate in the barley grain late in seed development [60]. This was in the period after the peak of photosynthesis in the pericarp when the activity of the pericarp specific NAD-malic enzyme [12] may have declined.

The expression of C_4_ genes in wheat spike bracts has been found to be greatly enhanced under water stress [61]. The levels of malate and oxaloacetic acid were also impacted by this stress. Enzymes of C_4_ photosynthesis and their metabolites were reported in wheat leaf bases and 42% of ^14^CO_2_ was incorporated into malate and aspartate [62]. Genes for each of the C_4_ specific enzymes have been located at different chromosomal locations to their C_3_ counterparts in the wheat genome [12,16].

Much less research has been conducted on photosynthesis in non-leaf tissues in other plant species. In soybean cotyledons, phosphoenolpyruvate (PEP) carboxylase is active early during the peak period of respiration followed by ribulose-1,5-biphosphate carboxylase/oxygenase rubisco later in germination [63]. However, the pathway has not been studied in detail in this system.

### 4.3. CAM

Crassulacean acid metabolism (CAM) [64] is similar to C_4_ in that a 4-carbon compound is the primary product of photosynthesis but the key feature is that this is separated with respect to time (in the dark and at lower temperatures so that carbon can be captured without large losses of water) from the decarboxylation and re-fixation by rubisco in the light. The specific presence of CAM in non-leaf tissues is not documented. However, many succulent plants from arid environments have a low surface area to volume ratio to avoid water loss and may use CAM or C_4_. They generally have an absence of thin leaf structures that could result in rapid water loss.

### 4.4. Carbon Isotope Discrimination and Non-Leaf Photosynthesis

The dark reactions of photosynthesis involve steps that show discrimination of carbon isotopes. This method has been widely used to evaluate the different modes of photosynthesis (C_3_, C_4,_ CAM) [17,65,66,67]. This works well in discriminating C_4_ and C_3_ steps in leaves because the carboxylating enzymes show different carbon isotope discrimination. However photosynthesis capturing carbon from respiration rather than the atmosphere requires many steps (including respiration) that involve carbon isotope discrimination, complicating interpretation in non-leaf tissue where this is the main source of carbon [17,18,65,66,67]. Studies of gas exchange in non-leaf tissues only measure net gas capture or production and do not detect turnover of carbon by respiration of carbon captured in the leaf and re-capture in non-leaf tissues. Carbon fixed in the leaf may be transported to the non-leaf tissue, respired, recaptured as a 4-carbon compound, decarboxylated and finally captured in a second round of C_3_ reactions. All of these steps may involve isotope discrimination. Changes during plant development allow an analysis of plant growth processes. Stable isotope analysis allows ecological research of present and past plant responses to environment [65].

## 5. Importance and Function of Non-Leaf Photosynthesis

**Figure 1:**
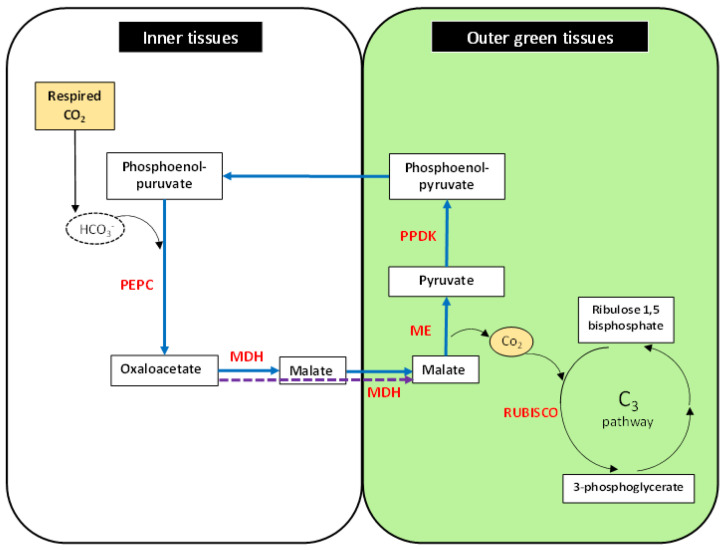
Photosynthesis in non-leaf tissues in outer and inner parts of the organ. Key reactions in inner tissues (without light) to capture respired carbon: PEPC, Phosphoenol-pyruvate carboxylase; MDH, malate dehydrogenase. Reactions in outer tissues (with light): ME, malic enzyme; RUBISCO, Ribulose-1,5-bisphosphate carboxylase/oxygenase; PPDK, Pyruvate orthophosphate dikinase.

The available evidence suggests that photosynthesis in non-leaf tissue may make an important contribution to plant growth and survival [1,50,68]. The leaf is a structure designed to use light to capture carbon from the atmosphere. Both light and atmospheric CO_2_ penetrates the short distance to the photosynthetic leaf cells. In many non-leaf tissues (especially seeds, stems, and thick petioles) light can only penetrate the outermost cell layers. In the deeper cells in these tissues, CO_2_ is not available from the atmosphere but is produced by respiration. In many systems [12,63] PEP carboxylase is active in capturing this carbon. The C_4_ photosynthesis pathway can only be completed in the outer layers of these organs or tissues where light can penetrate. A C_4_ malic enzyme is expressed specifically in the pericarp of the wheat grain [12]. This enzyme is known to have light-regulated expression [69]. This de-carboxylates malate to produce CO_2_ for rubisco in these highly photosynthetic cells. The cell layers outside the pericarp are clear allowing light penetration to the pericarp. This process is depicted in Figure 1 showing the reactions that are partitioned between the outer tissues exposed to light and the inner tissues that are in the dark and contributing net CO_2_ from respiration.

Stress has been shown to increase the expression of C_4_ rather than C_3_ specific genes in non-leaf plant systems. For example, this has been reported in alfalfa pod walls [48] and wheat [2]. This suggests that selection of plants with enhanced levels of expression of these traits [70] may be useful in breeding plants with greater stress tolerance. The deployment of genes encoding enhanced photosynthesis under stress requires an improved understanding of the specific function of some genes [60].

## 6. Evolutionary Considerations

Pathways of photosynthesis have evolved [71,72] many times independently in response to environment selection in different plant lineages. The C_4_ pathways have been identified as evolving at least 66 times in different plant groups [73]. This development is probably a response to a drying environment with lower concentrations of CO_2_ in the atmosphere at a time prior to the evolution of the C_4_ pathway. These evolutionary steps provides a mechanism for the plant to concentrate CO_2_ at the site of rubisco favouring the capture of CO_2_ from low atmospheric concentrations or low intra-cellular concentrations due to the plant limiting gas exchange to prevent water loss in hot or dry environmental conditions [74]. The pathways may have evolved to vary throughout the life of a plant as seasonal or environmental conditions vary. The evidence analyzed here suggests that these evolutionary steps may also have taken a different path in different plant organs or tissues [69]. Increased transcriptional abundance of the C_4_ specific gene copies has been identified as the initial step (with the same genes are co-opted across species), followed by anatomical and biochemical pre-conditioning in the evolution of the C4 pathway [75]. In a similar way, the distribution of crassulacean acid metabolism in orchids of Panama [76] has been reported to demonstrate the possibility for weak CAM prior to the evolution of strong CAM. A model has been proposed for the evolution of a weak C_4_ photosynthesis at first, followed by a post-emergence optimization process producing a strong C_4_ photosynthesis in leaves [64]. Reports of weak C4 in reproductive structures of maize [19,64] and sorghum [21] spikelets, when compared to that leaves, supports this hypothesis. The frequent evolution of C_4_ photosynthesis in different plant lineages [77] may have involved the development of intermediate processes on the path to full C_4_ photosynthesis in many cases. Evolution of these pathways in non-leaf tissues may have been driven more by the need to capture respired CO_2_ generated in deep tissues unable to conduct photosynthesis due to the lack of light penetration. Saving carbon losses would equate to the capture of the same amount of new carbon in the carbon balance of the plant.

## 7. Conclusions and Future Research Needs

Leaves are plant organs with a primary function of photosynthesis. However, other plant organs are also photosynthetic. Petioles, stems, seeds, and fruits are often green and clearly active in photosynthesis. Leaf anatomy supports photosynthesis by a C_3_ pathway and when Kranz anatomy is present C_4_ photosynthesis is partitioned into specialized cell types. However C_4_ photosynthesis may also be achieved by compartmentalization [78] within the cell without the presence of Kranz anatomy [78,79,80]. The pathways of metabolism in non-leaf tissues are less well studied. Separation of biochemical reactions deep within the organ and those on the surface with light penetration may be common in non-leaf tissues. A type of C_4_ photosynthesis has been reported in the petioles, stems, seeds, and fruits of C_3_ plants and variation in photosynthesis pathways in different parts of plants may be widespread [65]. Evidence for this is now found at the genome, transcriptome, and metabolite levels. Improved understanding of these processes may be critical in attempts to engineer plants with enhanced performance in future climates. Current gene-editing technology [81] should allow specific modification of steps in the pathways, either knockout the enzyme or enhancing expression. Those tools would allow evaluation of both the importance of these pathways for plant growth and performance and the details of the biochemical steps. This may allow the development of crops with the ability to use photosynthetic pathways flexibly and in different tissues in response to environmental signals [69]. The presence of weak CAM and C_4_, prior to the evolution of strong CAM and C_4_; and the model proposed for the evolution of a weak C_4_ or CAM that underwent a post-emergence optimization process to evolve a strong C_4_ or CAM, suggests a re-evaluation of the evolutionary timeline of the C_4_ pathway that would potentially address the reported time-lag [21] in the appearance of this pathway in response to low atmospheric CO_2_ levels. Enhanced photosynthetic efficiency and an ability of the plant to adapt photosynthesis to different environmental conditions would make an important contribution to the development of climate-resilient crop plants [82,83].

## Figures and Tables

**Table 1:** Studies of non-leaf photosynthesis in plants.

Tissue	Species	Reference
Root	Potato tuber	[9]
	*Vanda* (Orchidaceae)	[40]
	*Oncidium*	
	*Chiloschista*	
	*Arachnis*	
Trunk/Stem	Tobacco	[3]
	*Prunis illicifolia*	[13]
	*Umbellularia californica*	[13]
	*Arctostaphylos amnzanita*	[13]
	*Salix matsudana*	[14]
	*Clusia minor (Clusiaceae)*	[41]
	Cotton	[42]
Petiole	Tobacco, Celery	[3]
Flower	*Encelia spp* (Asteraceae)	[4]
	Giant ragweed (*Ambrosia trifida*)	[28]
	*Salsola spp (Chenopodiaceae)*	[43]
Seed	Soybean	[15]
	Barley (pericarp)	[44]
	Wheat	[12,45]
	Wheat (pericarp)	[46]
	Rice (ear)	[47]
	Alfalfa (pod walls)	[48]
	Castor (Rininus communis)	[49]
	Rape (Brassica napus) (embryo)	[6]
Fruit	Cucumber	[7]
	Tomato	[8]

**Table 2:** Examples of evidence for C4 pathways in non-leaf tissues of C3 plants.

Species	Stem/Petiole	Seed	Fruit
Tobacco	[3]		
Celery	[3]		
Wheat		[2,12]	
Barley		[44]	
Cucumber			[7]
